# Oligodendrocyte Progenitor Cells Become Regionally Diverse and Heterogeneous with Age

**DOI:** 10.1016/j.neuron.2018.12.020

**Published:** 2019-02-06

**Authors:** Sonia Olivia Spitzer, Sergey Sitnikov, Yasmine Kamen, Kimberley Anne Evans, Deborah Kronenberg-Versteeg, Sabine Dietmann, Omar de Faria, Sylvia Agathou, Ragnhildur Thóra Káradóttir

**Affiliations:** 1 Wellcome – Medical Research Council Cambridge Stem Cell Institute and Department of Veterinary Medicine, University of Cambridge, Cambridge, UK

**Keywords:** oligodendrocyte precursor cell, oligodendrocyte, myelin, differentiation, glutamate, neurotransmitter receptors, ion channels, glia, bioelectricity, electrophysiology

## Abstract

Oligodendrocyte progenitor cells (OPCs), which differentiate into myelinating oligodendrocytes during CNS development, are the main proliferative cells in the adult brain. OPCs are conventionally considered a homogeneous population, particularly with respect to their electrophysiological properties, but this has been debated. We show, by using single-cell electrophysiological recordings, that OPCs start out as a homogeneous population but become functionally heterogeneous, varying both within and between brain regions and with age. These electrophysiological changes in OPCs correlate with the differentiation potential of OPCs; thus, they may underlie the differentiational differences in OPCs between regions and, likewise, differentiation failure with age.

## Highlights

• Oligodendrocyte progenitor cells (OPCs) acquire ion channels with age
• OPCs become functionally heterogeneous both between brain regions and with age
• NMDARs disappear in non-myelinating regions but remain in actively myelinating areas
• Heterogeneity in OPC ion channel density indicates different functional states

## Graphical Abstract

**Figure:**
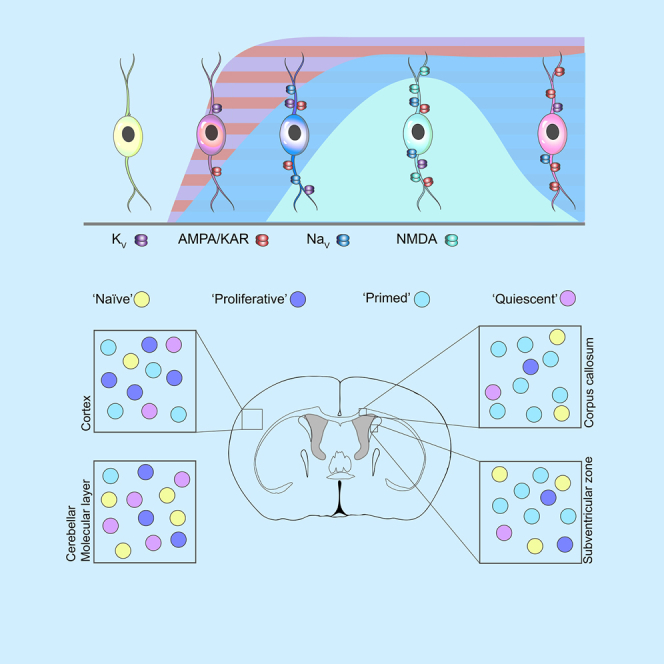

## Teaser

Spitzer et al. show that oligodendrocyte progenitor cells (OPCs) acquire ion channels and sensitivity to neuronal activity that differ between region and age. The onset and decline of ion channels follow developmental milestones. This heterogeneity indicates different functional states of OPCs.

## Introduction

Glial cells, astrocytes, oligodendrocytes, and microglia are conventionally thought of as homogeneous cell types; however, recent findings show that glial cells, like neurons, have a different transcriptome and proteome depending on the brain region and with age and that the glial cell transcriptome, but not the neuronal one, is mostly altered by aging (Soreq et al., 2017). How these changes relate to cellular function is unclear, and the functional implications of glial cell heterogeneity and age-related brain changes are yet to be understood.

The fourth type of glial cell, the oligodendrocyte progenitor cell (OPC), which expresses the proteoglycan NG2, is widely distributed throughout the adult brain and is the main proliferative cell present in the adult CNS (Dawson et al., 2003). Its function in the adult CNS is relatively unclear. During development, OPCs give rise to oligodendrocytes that generate myelin, which ensures fast signal transmission and provides metabolic support to axons (Nave, 2010, Saab et al., 2016). It is becoming evident that, in young adults, OPCs are needed for motor learning (McKenzie et al., 2014, Sampaio-Baptista et al., 2013, Xiao et al., 2016), myelin maintenance (Young et al., 2013), and myelin regeneration (Zawadzka et al., 2010). With age, however, motor learning and myelin regeneration decline (Huang et al., 2011, Ruckh et al., 2012), and white matter lesions accumulate (Habes et al., 2016), indicating a reduced potential of the OPCs for *de novo* myelination, maintenance, and regeneration.

Growing evidence, both *in vitro* and *in vivo*, shows that neuronal activity and glutamate signaling are regulatory signals for myelination and remyelination in young adults (de Faria et al., 2018, Gautier et al., 2015, Krasnow et al., 2018, Lundgaard et al., 2013, Mitew et al., 2018). OPCs have the capacity to sense changes in neuronal activity and glutamate release because OPCs express glutamate receptors and receive depolarizing synaptic inputs from axons in both white and gray matter (Bergles et al., 2000, Káradóttir et al., 2005, Káradóttir et al., 2008, Kukley et al., 2007, Spitzer et al., 2016, Ziskin et al., 2007). These electrophysiological properties, along with the expression of voltage-gated ion channels such as tetrodotoxin (TTX)-sensitive voltage-gated sodium channels, have become a defining feature of OPCs (Clarke et al., 2012, De Biase et al., 2010). However, not all OPCs express these electrophysiological properties (Chittajallu et al., 2004, Káradóttir et al., 2008). Whether this heterogeneity has a functional significance or whether it is solely a transitional stage prior to differentiation is debated (Clarke et al., 2012, De Biase et al., 2010).

Whether OPCs are a heterogeneous cell population is controversial. On one hand, depletion and functional studies have indicated that forebrain OPCs, despite arising from three different origins in subsequent waves during early development, are functionally similar (Clarke et al., 2012, Tripathi et al., 2011). Similarly, electrophysiological studies of OPCs in the hippocampus and corpus callosum (CC) have reported OPCs to be homogeneous (Clarke et al., 2012, De Biase et al., 2010). On the other hand, OPCs have been shown to respond differently to growth factors (Mason and Goldman, 2002) and cytokines (Lentferink et al., 2018), and they have differential transcriptional profiles (Marques et al., 2018). Furthermore, depending on the region, OPCs differ in their capability to differentiate into myelinating oligodendrocytes. For example, OPCs from the CC differentiate into myelinating oligodendrocytes more efficiently than OPCs taken from the cortex (CTX; gray matter) (Viganò et al., 2013). In line with this, the electrophysiological properties of OPCs reportedly differ between gray and white matter OPCs (Chittajallu et al., 2004) despite having the same developmental origin.

Hence, we asked whether the electrophysiological properties of OPCs change with age and brain region as transcriptional studies have demonstrated for other types of glial cells, altering their capability to monitor and respond to neuronal activity. To address this, we used single-cell electrophysiological recordings in heterozygous NG2-EYFP (enhanced yellow fluorescent protein) knockin mice, where the expression of the reporter gene is regulated according to the endogenous NG2 gene, allowing unbiased sampling of this population. We report that OPCs start out as a homogeneous population but become functionally heterogeneous, varying both within and between brain regions and age. These electrophysiological changes correlate with the differentiation potential of OPCs.

## Results

### OPCs Acquire Functional Voltage-Gated Ion Channels at Different Developmental Time Points

**Figure 1:**
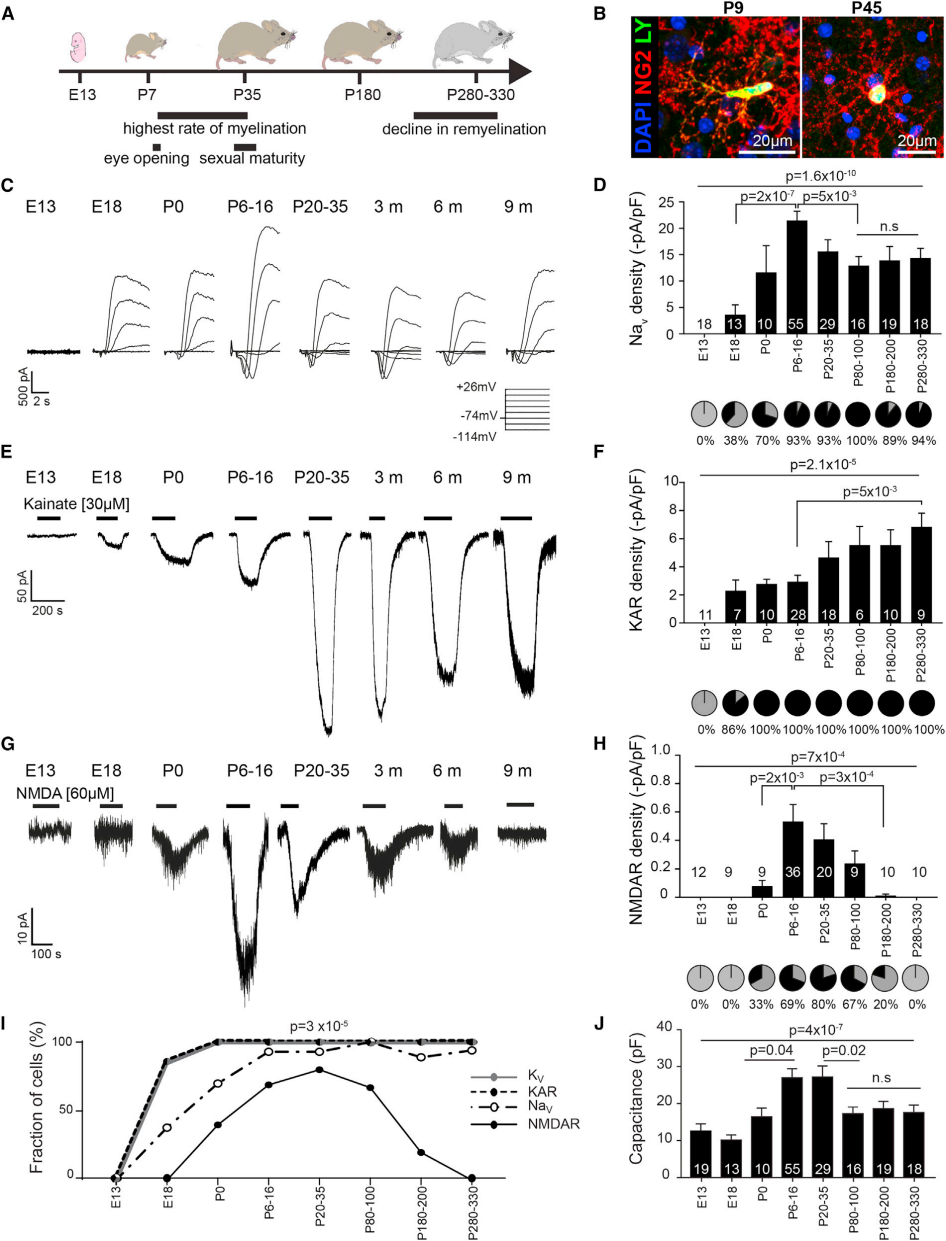
OPCs Acquire Functional Ion Channels at Different Developmental Time Points (A) Schematic diagram of the mouse developmental timeline, indicating developmental and myelination-related milestones over the period studied. (B) Cells were selected by their EYFP expression (NG2-EYFP knockin mice). During whole-cell patch-clamp recording, OPCs were dye-filled with Lucifer Yellow (LY, green) and post hoc-labeled for NG2 (red). Scale bar, 20 μm. (C) Leak-subtracted traces of voltage-gated sodium currents (Na_V_) in response to 20-mV steps from a holding potential of −74 mV (inset, voltage pulses from −114 to +26 mV) in OPCs from E13 to 9-month-old-mice. (D) Na_V_ densities were significantly different between age groups (p = 1.6 × 10^−10^). Na_V_ densities peak at P6–P16 (at the time when myelination is at its highest rate; A). The proportion (pie charts) of OPCs with (black) or without (gray) detectable Na_V_ currents differed (p < 1 × 10^−15^, χ^2^) with age. (E) Kainate (30 μM)-evoked currents in OPCs from E13 to 9-month-old-mice. (F) The density of AMPA/kainate receptors (KARs) increased steadily with age (p = 2.1 × 10^−5^, ANOVA), and the proportion (pie charts) of OPCs with (black) detectable KA-evoked currents increased (p < 1 × 10^−15^, χ^2^) until after birth, when all OPCs had detectable KA-evoked currents. (G) NMDA (60 μM)-evoked currents in OPCs from E13 to 9-month-old-mice. (H) The density of NMDA receptors (NMDARs) in OPCs changed significantly with age (p = 7 × 10^−4^, ANOVA). Similarly, the proportion (pie charts) of OPCs with (black) detectable NMDA (60 μM)-evoked currents changed with age (p < 1 × 10^−15^, χ^2^). Both current density and OPCs with detectable currents peaked during P6–P35, at the time when myelination is at its highest rate, and declined until becoming undetectable (gray). (I) The fraction of OPCs with detectable voltage-gated potassium (K_V_) or Na_V_ channels and with detectable KAR-evoked and NMDA receptor (NMDAR)-evoked currents across the lifespan. K_V_ and KA-evoked currents are first detected in OPCs, followed by Na_V_; all three remain present in the majority of postnatal OPCs throughout life. In contrast, NMDA-evoked currents are detected last, and only during the period of highest myelination are NMDARs detected in the majority of OPCs. At later ages, NMDARs become undetectable. K_V_, p < 1 × 10^−15^; Na_V_, p < 1 × 10^−15^, χ^2^; KAR, p < 1 × 10^−15^, χ^2^; NMDA, p < 1 × 10^−15^, χ^2^. The onset of detection between receptors differs (p = 4.3 × 10^−6^), and the fraction of OPCs with detectable currents differs across age (p = 2.7 × 10^−5^). (J) OPC capacitance peaks with myelination rate and then declines back to perinatal levels. The numbers shown on graphs represent the number of whole-cell patched OPCs from 2–21 animals. Top p values are from ANOVA analyses, whereas bottom p values are from post hoc Holm-Bonferroni analyses. Data are shown ± SEM.

To investigate the electrophysiological membrane properties of OPCs from first appearance to old age, we used heterozygous NG2-EYFP knockin mice (NG2-EYFP); in these mice, all parenchymal EYFP^+^ cells are Olig2- and NG2-positive throughout life, indicating that EYFP expression tightly follows expression of the NG2 protein (Karram et al., 2008; Figure 1B). We voltage-clamped OPCs at embryonic day 13 (E13) because mouse forebrain OPCs first appear at E12.5 (Kessaris et al., 2006); then at E18, when the second wave (E15.5) is established; at postnatal day 0 (P0), which coincides with the third wave (Kessaris et al., 2006); then weekly for the first month, when myelination rates are the highest (Hamano et al., 1998); and then every 2 to 3 months until P330, when myelination efficacy has declined (Huang et al., 2011, Tripathi et al., 2017; Figure 1A).

A supposed defining feature of OPCs is the presence of voltage-gated sodium channels (Na_V_), a stereotypic transient inward current that is evoked upon depolarization beyond −50 mV. This depolarization-evoked transient inward current was absent in OPCs at E13. In fact, at this time, no voltage-gated currents were detected in OPCs. We first detected a depolarization-evoked transient inward current, known to be TTX-sensitive Na_V_ (De Biase et al., 2010, Káradóttir et al., 2008), and outward currents known to represent voltage-gated potassium channel (K_V_) currents, in a subset of OPCs at E18. Notably, K_V_ currents were detected in 85% of cells, whereas Na_V_ were detected in only 38% of OPCs at E18 (p = 0.04), indicating that K_V_ channel expression starts before Na_V_ expression. Although both the proportion of OPCs with functional Na_V_ channels and their density sharply increased after birth, the proportion of OPCs with Na_V_ channel expression lagged behind K_V_ channels (Figure 1I). The peak Na_V_ channel density coincided with the start of myelination (21.5 ± 1.7 pA/pF, p = 1.6 × 10^−10^) and was detected in over 90% of OPCs recorded from the second postnatal week (Hamano et al., 1998). The density of Na_V_ channels began to decline after the first month to a lower steady level (13.8 ± 1.2 pA/pF, p = 5 × 10^−3^) following the end of the peak period of developmental myelination (Hamano et al., 1998; Figures 1C, 1D, and 1I).

Despite the decline in Na_V_ density with age, the majority, but not all, of postnatal OPCs had detectable Na_V_ currents (Figures 1D and 1I). OPCs without Na_V_ had a compact high membrane resistance, similar to that of OPCs with Na_V_ (p = 0.54), but not a low membrane resistance, as is more reminiscent of OPCs that have started to differentiate (De Biase et al., 2010). Cell capacitance, a measure of membrane surface area, significantly changed with age (p = 4 × 10^−7^), starting low, peaking at the time when the myelination rate is highest in the forebrain, and then declining after the first month to a level similar to that of prenatal cells (Figure 1J). None of these properties differed between male and female animals (Figures S1A–S1C).

These data show that newly formed OPCs acquire voltage-gated ion channels at different rates and that the density of voltage-gated ion channels, and thus OPC excitability, changes throughout the life of the animals. The timing of these changes, intriguingly, aligns with key milestones of myelination and development.

### Glutamate Receptor Subtypes Appear at Different Developmental Time Points

To address when OPCs acquire functional glutamate receptors, we voltage-clamped OPCs from E13–P330 as before. We first detected kainate-evoked currents (30 μM; activates both α-amino-3-hydroxy-5-methyl-4-isoxazolepropionic acid [AMPA] and kainate [KA] receptors) in a majority of OPCs at E18 (86%; Figures 1E, 1F, and 1I), coinciding with the appearance of K_V_. At this time point, N-methyl-D-aspartate (NMDA; 60 μM)-evoked currents were undetectable (Figures 1G–1I). When myelination in the forebrain is at its highest rate (P6–P35; Hamano et al., 1998), NMDA receptor (NMDAR) density peaked, coinciding with peak Na_V_ density, and, at this time, the proportion of OPCs with NMDARs was the highest, at ∼80% (Figures 1H and 1I). In contrast to NMDA, KA-evoked currents were detected in nearly all OPCs after birth (Figure 1E, 1F, and 1I). AMPA/KA receptor (KAR) densities gradually increased with age (p = 2.1 × 10^−5^; Figures 1E and 1F). Following sexual maturity, NMDAR density began to decrease, and NMDA-evoked currents were barely detectable by 6 months (0.006 ± 0.005 pA/pF, p = 3 × 10^−4^; Figures 1G–1I) and completely undetectable at 9 months, a time when remyelination potential has declined (Huang et al., 2011) and addition of new myelinating oligodendrocytes in the CC has ceased (Tripathi et al., 2017). The glutamate receptor density in OPCs did not, however, differ between female and male animals (Figures S1D–S1F). Spontaneous synaptic-like inputs became prominent at the end of the first week (p = 0.008) and remained comparable throughout life (0.02 ± 0.008 Hz, p = 0.2). These data show that OPCs acquire functional glutamate receptors at different rates and that their density peaks at different ages. In particular, NMDAR density and the proportion of OPCs with NMDARs peaked at the time of myelination. Importantly, although OPCs receive synaptic inputs at similar frequencies throughout life, their response to these inputs is likely to vary with age, in line with altered glutamate receptor expression.

### OPC Molecular Signatures Differ with Age

**Figure 2:**
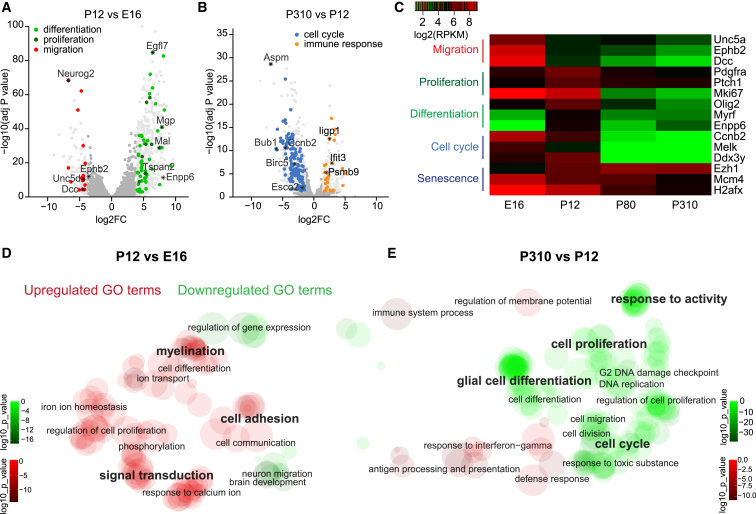
OPC Molecular Signatures Change with Age (A) Volcano plot showing differential expression of significantly altered genes for P12 compared with E16. Light gray circles indicate genes that are more than 4-fold increased between P12 and E16. Significantly altered gene ontology (GO) terms are highlighted (red, migration; dark green, proliferation; light green, differentiation). ^∗^selected genes of interest. (B) As for (A) but for P310 compared with P12; all circles denote significantly altered genes. Light gray circles represent genes that are more than 1.5-fold increased. A selection of significantly altered GO terms are highlighted (blue, cell cycle; orange, immune response). ^∗^selected genes of interest. (C) Heatmap of genes related to migration, proliferation, differentiation, cell cycle, and senescence across ages. Reads per kilobase million (log2(RPKM)) are plotted. (D) REVIGO semantic clustering visualization of GO analysis, showing all significantly altered terms at P12 compared with E16. The circle size approximately represents the number of genes within a GO term; the color intensity reflects the log10 p value. GO terms of particular interest are highlighted. (E) As for (D) but showing the GO terms at P310 compared with P12.

The observed changes in ion channel and glutamate receptor densities in OPCs with age, intriguingly, align with milestones of myelination and development. Thus, to test whether the molecular signatures of key biological properties in OPCs also change with age, we performed bulk RNA sequencing (RNA-seq) of OPCs isolated by PDGFRα magnetic activated cell sorting (MACS; enriching for OPCs expressing PDGFRα) at different ages: E16, P12, P80, and P310. We observed that embryonic OPCs had molecular signatures of migrating cells (e.g., *Dcc* and *Ephb2*), significantly more so compared with OPCs from P12 or older mice. However, at P12, OPCs had stronger signatures of proliferating (e.g., *Pdgfra*, *Ptch1*, and *Mki67*) and committed OPCs (*Myrf* and *Enpp6*) and upregulated signal transduction but a reduced migratory molecular signature (Figures 2A, 2C, and 2D). Both molecular signatures of differentiation and proliferation decreased from P12 to P80 and further decreased in old OPCs. Between P12 and P80, there is a significant decrease in molecular signatures for cell cycle regulation (e.g., *Mki67*, *H2afx*, and *Ccnb2*), differentiation, and metabolism at the time when myelination starts to decline (Figure 2C). OPCs from older animals further downregulate cell cycle regulatory and differentiation genes, concomitant with upregulation of pro-quiescence genes and downregulation of genes regulating anti-quiescence, sensing neuronal activity, stem cell maintenance, and transcription (Figures 2B, 2C, and 2E).

The gene expression of Na_V_ (α subunits *Scn3a* and *Scn7a*, and β auxiliary subunits *Scn1b*, *Scn3b*, and *Scn4b*) and NMDAR subunits (*Grin1*, *Grin2a*, *Grin 2C*, and *Grin3a*) significantly increased from embryonic to postnatal OPCs, and subsequently all remained constant to old age, with the exception of *Grin3a* and *Scn3b*. However, AMPAR and KAR subunits, apart from *Grik3*, remained constant from E16 to old age (Figures S2A–S2C; Table S1). These results reflect that Na_V_, NMDAR, and AMPA/KAR protein expression is post-transcriptionally and/or post-translationally regulated in OPCs.

### OPC Proliferation Decreases with Age in Line with Na_V_ Density

**Figure 3:**
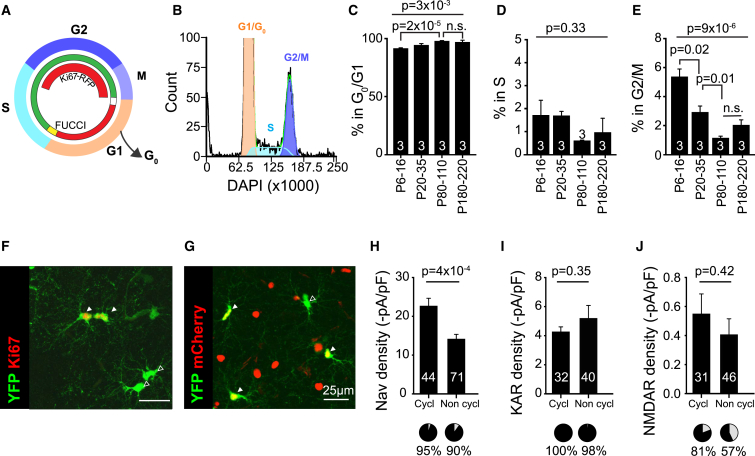
OPC Cell-Cycle Changes with Age and Na_V_ Channel density (A) Schematic diagram showing the fluorescent labeling seen at different points in the cell cycle for fluorescent ubiquitination-based cell-cycle indicator (FUCCI) and Ki67-RFP mice. (B) Representative image of flow cytometry analysis of relative DAPI intensity in OPCs, with the color coding of peaks representing different cell cycle stages. (C–E) The percentage of OPCs in G_0_/G1 (C), S phase (D), and G2/M phase (E) as measured by flow cytometry. Numbers on graphs represent the number of animals used per time point. (F) Ki67-RFP:NG2-EYFP cortical section showing yellow fluorescent protein (YFP)-positive (green) OPCs with (white arrowheads, in S/G2/M) and without (black filled arrowheads, in G_0_/G1) Ki67 (red) expression. Scale bar, 25 μm. (G) FUCCI:NG2-EYFP cortical section showing YFP^+^ OPCs with (white arrowheads, in G_0_/G1) and without (black filled arrowheads, in S/G2/M) mCherry labeling. Scale bar, 25 μm. (H) Na_V_ densities were significantly higher in cycling OPCs compared with non-cycling (cycl, cycling [in S/G2/M]; non cycl, non-cycling [in G_0_/G1]), whereas the proportion of OPCs with detectable currents did not change (pie chart, p = 0.50, χ^2^). (I and J) Neither (I) KAR nor (J) NMDA densities differed between cycling and non-cycling OPCs, nor did the proportions of cells responding (pie charts; KAR, p = 0.86; NMDAR, p = 0.051; χ^2^). Numbers on graphs represent the number of cell recorded from 18 animals. The p values are from Student’s t tests (H–J) or one-way ANOVA (C–E, top), followed by Holm-Bonferroni post hoc test (bottom). Data are shown ± SEM.

Given the clear change in cell cycle regulatory signatures in older OPCs, we investigated age-related cell cycle changes in OPCs with flow cytometry (Figures 3A, 3B, and S2E–S2G). We identified that the proportion of OPCs in G_0_/G1 and G2/M phase changed with age (p = 0.003 and p = 9 × 10^−6^; Figures 3C and 3E), but not the proportion of OPCs in S phase (p = 0.33; Figure 3D). The proportion of OPCs in G2/M phase peaked around P12 (Figure 3E), reflecting the high proliferative state at this time point, which coincided with the peak Na_V_ density (Figures 1C and 1D) and expression of proliferative genes (Figure 2C), and plateaued at P80, a time when Na_V_ density plateaued and cell cycle genes were downregulated (Figures 2C and 2E). The proportion of OPCs in G_0_/G1 phase gradually increased with age (Figure 3C), indicative of a gradual lengthening of G1 phase or an accumulation of OPCs entering G_0_ phase. The gradual increase in G_0_/G1 resembled the gradual increase in AMPA/KAR densities.

To test whether ion channel expression changes with the cell cycle, we crossed NG2-EYFP mice with either Fucci2a mice, in which mCherry is expressed during G_0_/G1 phase (Mort et al., 2014; Figures 3A and 3G), or Ki67-RFP mice, in which the reporter protein RFP is expressed during G2/M phase (Basak et al., 2014; Figures 3A and 3F). OPCs in an active cell cycle (cycling, Ki67-RFP^+^ or Fucci-mCherry^−^) have a 1.7-fold higher density of Na_V_ than OPCs in G_0_/G1phase (non-cycling, Ki67-RFP^−^ or Fucci-mCherry^+^; Figure 3H; p = 4 × 10^−4^). There was no change detected in either NMDAR or AMPA/KAR densities between G2/M phase and G_0_/G1 phase of the cell cycle (NMDAR, p = 0.42; AMPA/KAR, p = 0.35; Figures 3I and 3J) or in the proportion of OPCs with detectable evoked currents (NMDAR, p = 0.051; AMPA/KAR, p = 0.86). Neither mouse line can discriminate between OPCs in G1 and G_0_ phases of the cell cycle. However, if ion channel densities in the G1 phase differ from G_0_, then an increased heterogeneity would be expected in the recordings from non-cycling OPCs (G_0_/G1 phase) compared with cycling OPCs (G2/M phase). To test for heterogeneity, we analyzed the variance in the dataset between non-cycling OPCs and cycling OPCs. The variance in the density of Na_V_ channels and NMDARs was identical between both states (Na_V_, p = 0.08; NMDAR, p = 0.87), whereas the variance in AMPA/KAR densities differed greatly (p = 5 × 10^−8^), implying that AMPA/KAR densities in OPCs might differ between G1 and G_0_ phase of the cell cycle.

OPC cell cycle time has been reported to differ between the CC and CTX and with age (Young et al., 2013). We investigated the proportion of OPCs labeled with the cell cycle protein Ki67 in brain slices from NG2-EYFP mice. In line with the RNA-seq and flow data, the proportion of proliferating OPCs both in the CC and CTX sharply decreased after the first postnatal weeks (Figures S2H–S2J). In the CC, the proportion of Ki67^+^ OPCs reached a constant level by the end of the first month, whereas in the CTX, the proportion of proliferating OPCs gradually decreased with age (Figures S2I and S2J). On average, the proportion of proliferating OPCs was higher in the CC compared with the CTX; however, at a very old age, the proportion of Ki67^+^ OPCs between the CC and CTX became nearly identical (Figures S2I and S2J).

### Gray and White Matter OPCs Diverge with Age

**Figure 4:**
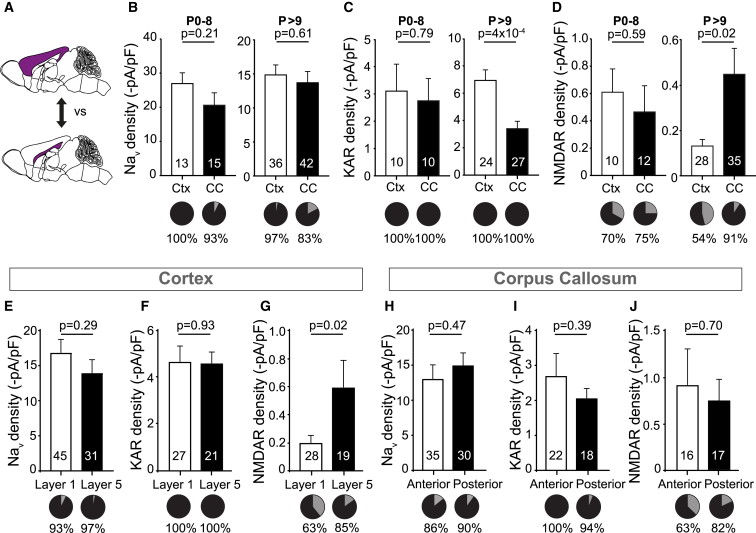
OPCs Become Regionally Diverse after the First Postnatal Week (A) OPCs were whole-cell patch-clamped in the corpus callosum or the cortex, as highlighted in the schematic diagram in purple. (B) Na_V_ densities (bar graph) and the proportion of OPCs (pie charts) with detectable Na_V_ currents (black) do not differ between the corpus callosum (CC) or the cortex (CTX), neither during the first postnatal week (p = 0.21, p = 0.94, χ^2^) nor thereafter (p = 0.61, p = 0.10, χ^2^). (C) AMPA/KAR densities did not differ between OPCs in the white matter (CC) or gray matter (CTX) at birth (p = 0.79, left) but became significantly different after P8 (p = 4 × 10^−4^). Throughout postnatal life, KA (30 μM) evoked currents in OPCs (pie charts underneath the bar graph, with [black] or without [gray] detectable evoked currents). (D) NMDAR densities and the proportion of OPCs with detectable NMDA (60 μM)-evoked currents (pie charts) were not different between OPCs in the CC or the CTX at birth (p = 0.59, p = 0.83, χ^2^) but became significantly different after P8 (p = 0.02, p = 1.7x10^−3^, χ^2^), with reduced NMDA-evoked currents in OPCs in the CTX. (E and F) Neither Na_V_ (E) nor KAR (F) densities nor the proportion of cells with detectable Na_V_ (p = 0.1, χ^2^) (E) or KA-evoked currents (p > 0.99, χ^2^) (F) differed between layer 1 and layer 5 of the CTX. (G) NMDAR densities were higher in layer 5 of the CTX compared with layer 1, but the proportion of OPCs with detectable NMDA-evoked currents did not differ between the two layers (p = 0.1, χ^2^). (H–J) There was no difference detected between the anterior or posterior CC in receptor densities or the proportion of cells responding to (H) Na_V_ (p = 0.88, χ^2^), (I) KAR (p = 0.88, χ^2^), and (J) NMDAR (p = 0.37, χ^2^). Numbers shown on bar graphs represent cell numbers from 5–20 animals. Data are shown ± SEM. The p values are from Student’s t tests.

OPCs have been reported to differ in both myelination potential (Viganò et al., 2013) and ion channel expression (Chittajallu et al., 2004, Káradóttir et al., 2008) between the CTX and CC. Hence, we investigated when OPCs in these regions begin to differ. Recording from OPCs in both areas during the first postnatal week, in gray (CTX) and white (CC) matter, we found that OPCs are almost indistinguishable between regions with respect to Na_V_ (p = 0.21), AMPA/KAR (p = 0.79), and NMDAR (p = 0.59) densities and frequency of synaptic inputs (p = 0.12). Hence, at this time, OPCs have a similar sensitivity to neuronal activity. However, after the first postnatal week, OPCs diverged and varied significantly between gray and white matter, particularly in regard to AMPA/KAR (p = 4 × 10^−4^) and NMDAR (p = 0.02) densities (Figures 4A–4D). Similarly, OPCs in the CTX diverged even further, as NMDAR (p = 0.02) densities differed between cortical layers 5/6, which is a myelinated area in the CTX, and layer 1, which is nearly unmyelinated; Na_V_ channels (p = 0.29) and AMPA/KAR (p = 0.93) densities were unchanged between the two cortical layers (Figures 4E–4G). The white matter, on the other hand, is more homogeneous; no differences were identified in Na_V_ channels (p = 0.47), AMPA/KAR (p = 0.39), and NMDAR (p = 0.70) densities or proportions (Na_V_, p = 0.88; AMPA/KAR, p = 082; NMDAR, p = 0.79) in OPCs located in either the anterior or posterior CC (Figures 4H–4J). These results indicate that the local environment affects OPC ion channel expression and, potentially, myelination potential.

To test whether the microenvironment can alter OPC electrophysiological properties, we cultured neonatal OPCs before their regional specification under different culture medium conditions. Glutamate receptor, NMDAR, and Na_V_ densities were sensitive to the constituents of the medium (Figure S3). The effects of serum were reversible by neuronal conditioned medium in neonatal OPCs. These data show that altering the milieu around neonatal OPCs is sufficient to alter their functional ion channel expression.

### The Age-Related Differentiation Potential of OPCs Is Not Reversed by an Altered Environment

**Figure 5:**
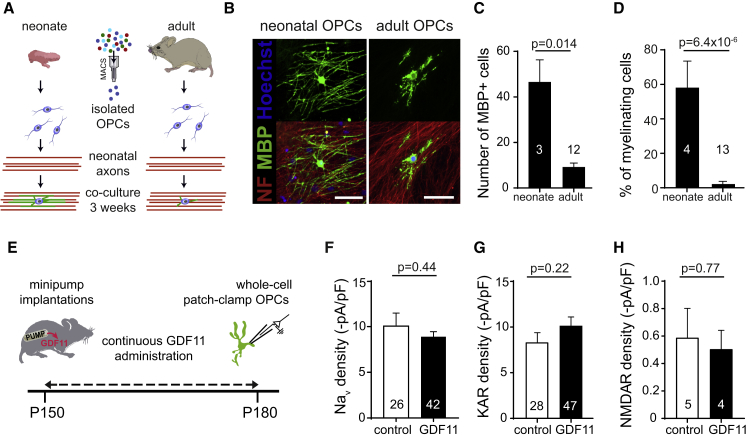
The Age-Related Reduction in Myelination Potential and Ion Channel Expression in OPCs Is Not Reversed by an Altered Environment (A) Schematic of the generation of myelinating OPC-DRG co-cultures. OPCs were isolated by magnetic-activated cell sorting (MACS) from either neonate or adult mice and plated onto neonatal DRG neurons. (B) High-magnification views of a myelinating neonatal oligodendrocyte (top, left) with MBP (green) expressed in processes wrapping axons expressing neurofilament (NF) 160/200 (NF, red, bottom, left), and of an MBP expressing non-myelinating oligodendrocytes from old animals (top, right) where the MBP+ processes are not aligned with axons (bottom, right). Scale bar, 50 μm. (C and D) Quantification of differentiated oligodendrocytes (MBP^+^) in co-cultures comprising neonatal dorsal root ganglion neurons and neonatal or aged OPCs; neonatal OPCs produced more (C) MBP^+^ cells per coverslip and a higher fraction of (D) myelinating cells (right). Numbers represent the number of experiments. (E) Schematic diagram of delivery of GDF11 via minipumps implanted at P150, allowing continuous i.p. infusion of GDF11 until P180, when OPCs were whole-cell patch-clamped. (F–H) Ion channel densities were not significantly different between GDF11 and control-treated animals: (F) Na_V_ densities (p = 0.44), (G) KAR densities (p = 0.22), and (H) NMDAR densities (p = 0.77). Numbers shown on bar graphs represent cell numbers recorded from 2–3 animals. Data are shown ± SEM. The p values are from Student’s t tests.

Age-related changes in the systemic milieu have been shown to lead to a decrease in ion channel function in hippocampal neurons, similar to the changes we detect in OPCs, and are linked to declining cognitive function. Similarly, the differentiation and myelination potential of OPCs also decreases with age. Parabiosis of young blood can reverse both of these age-related changes (Ruckh et al., 2012, Villeda et al., 2011, Villeda et al., 2014). Therefore, we tested whether OPCs isolated from old mice, when the NMDARs had declined *in vivo*, could be reverted to have a similar myelination potential as neonatal OPCs when cultured in myelin-promoting medium for 3 weeks with neonatal dorsal root ganglion (DRG) neurons, mimicking youth (Figure 5A). The neonatal OPCs differentiated and myelinated the DRG axons, but only a small fraction of the adult OPCs differentiated, and only into non-myelinating MBP-positive cells (Figures 5B–5D; p = 6.4 × 10^−6^). Administration of growth and differentiation factor (GDF) 11 has been shown to recapitulate some of the rejuvenative effects of young blood in mice, although it has not yet been tested for myelination (Katsimpardi et al., 2014, Sinha et al., 2014). We thus tested whether the changes we detected in electrophysiological properties of OPCs could be reverted by *in vivo* administration of GDF11 for 4 weeks (intraperitoneally [i.p.], 0.1 mg/kg) from P150, when Na_V_ and NMDAR density has declined. No differences were detected in Na_V_ channel, AMPA/KAR, or NMDAR densities (Figures 5E–5H; Na_V_, p = 0.44; KA, p = 0.22; NMDA, p = 0.77) compared with saline administration. These data indicate that when OPC properties have changed, as occurs with maturation, ion channel expression and myelination potential do not easily revert to that of neonatal OPCs.

### OPCs Become Heterogeneous between and within Regions

**Figure 6:**
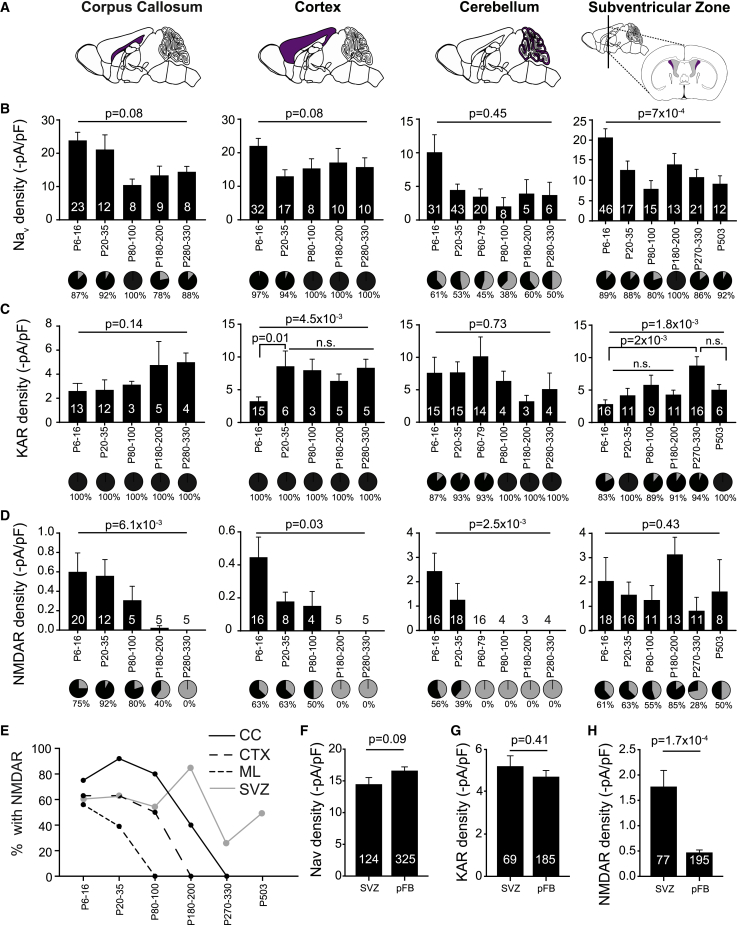
Ion Channel Densities in OPCs Change Differently across the Lifespan in the CC, CTX, Cerebellum, and Subventricular Zone (A) Illustration of the brain areas (purple) where OPCs were whole-cell patch-clamped: CC, a highly myelinated region; CTX, a lightly myelinated region; cerebellar molecular layer (ML), a region that is never myelinated in mice; and subventricular zone (SVZ), an area that provides a continuous supply of myelinogenic OPCs throughout life. (B) Na_V_ densities (bar graph) did not change across postnatal age in the CC (left), CTX (center left), or ML (center right) but did change in the SVZ (right). The proportion of OPCs with detectable Na_V_ (black) changed throughout postnatal life in the CC (p = 3.1 × 10^−5^, χ^2^), the CTX (p = 2.6 × 10^−3^, χ^2^), and the ML (p = 6.5 × 10^−3^, χ^2^), but not the SVZ (p = 0.7, χ^2^). (C) AMPA/KAR densities increased with age in the CTX and the SVZ but remained stable in other areas throughout life. Throughout postnatal life, KA (30 μM) evoked currents in all OPCs except in the ML, where, in the first 3 months of life, a small proportion of OPCs had no detectable KA-evoked currents, and in the SVZ, where OPCs with no KA-evoked response could be found throughout life. The proportion of OPCs with kainite-evoked currents only changed with age in the hindbrain (CC, p = 1, χ^2^; CTX, p = 1, χ^2^; ML, p = 2.5 × 10^−6^, χ^2^; SVZ, p = 0.61, χ^2^). (D) NMDAR densities significantly declined in all areas except the SVZ across postnatal life, although with a different rate in each area. The proportion of OPCs with detectable NMDA (60 μM)-evoked currents (pie charts) were significantly different across age in all areas except the SVZ, recorded from the following: CC, p < 1 × 10^−15^, χ^2^; CTX, p < 1 × 10^−15^, χ^2^; ML, p < 1 × 10^−15^, χ^2^; SVZ, p = 0.62, χ^2^. (E) The fraction of postnatal OPCs with detectable NMDA-evoked current in the CC), CTX, ML, and SVZ. NMDARs became undetectable at different times in each region but not in the SVZ (p = 0.003, two-way ANOVA, comparison between areas). (F and G) Neither (F) Na_V_ nor (G) KAR densities differed between parenchymal forebrain OPCs (pFB) and SVZ OPCs. (H) NMDAR density was higher in SVZ OPCs compared with pFB OPCs. Numbers shown on bar graphs represent cell numbers from 1–13 animals. Data are shown ± SEM. Top p values are from ANOVA analyses, and bottom p values are from Holm-Bonferroni post hoc tests.

Next we addressed whether the changes in ion channel expression we identified differ between white matter (CC) and a gray matter region that has some myelination (CTX) or a gray matter region that is never myelinated (molecular layer of the cerebellum [ML]) and the subventricular zone (SVZ), an area that provides a continuous supply of myelinogenic OPCs throughout life (Menn et al., 2006; Figure 6A). At P7, OPCs in all regions tested had detectable Na_V_, AMPA/KAR, and NMDAR currents (Figures 6B–6E). There was a clear divergence in expression patterns between gray and white matter after the first postnatal weeks. NMDA-evoked currents rapidly disappeared in the ML OPCs after the first month, whereas NMDA-evoked currents in the CTX declined slower and became undetectable just after 3.5 months (p = 5 × 10^−3^; Figure 6E). In contrast, OPCs in the CC showed a slower decline in NMDAR density and slightly higher NMDAR densities than those in the CTX (Figure 6D), and a larger portion of CC OPCs, ∼80% on average, had functional NMDARs compared with around half of the OPCs in the CTX (p = 3 × 10^−6^) and ML (p = 9 × 10^−14^; Figures 6D and 6E). In contrast to the parenchymal regions, NMDAR densities and the proportion of OPCs with NMDA-evoked currents remained unchanged throughout life in the SVZ (p = 0.43, p = 0.62) and were even detected in animals up to P503 (Figures 6D, 6E, 6H). The density of NMDARs in OPC in the SVZ was ∼4 fold higher than in parenchymal OPCs (p = 1.7 × 10^−4^). Moreover, there was much greater variability (p < 1 × 10^−15^) in the NMDA-evoked currents in the SVZ compared with parenchymal OPCs, presumably indicating continuous cycles of early-born and old OPCs in the SVZ (Figure S4E).

KA evoked currents in nearly all postnatal OPCs regardless of age and region, except in the SVZ, where there was a higher proportion of OPCs with no detectable KA-evoked currents (p = 0.024; Figure 6C). Most OPCs without detectable KA-evoked currents also lacked detectable Na_V_, reminiscent of early embryonic OPCs (Figures 1C–1F). The AMPA/KA receptor densities tended to increase with the decline in NMDAR density, particularly evident in the CTX, where AMPA/KAR densities sharply increased at the same time as NMDAR density declined. Conversely, the density of KA receptors in OPCs in and around the SVZ only gradually increased and peaked late, at P270–P330 (p = 2 × 10^−3^), whereas in the CTX, the AMPA/KAR density peaked at P20–P35 (p = 0.01; Figure 6C).

These data show that OPCs are heterogeneous, varying mostly between regions and with age. The SVZ stands out as the area with the greatest heterogeneity within each time point but the most homogeneous across age. Intriguingly, NMDAR expression seems to be highly regulated in OPCs and may provide an explanation for why OPCs in the gray matter show less myelination potential than white matter OPCs (Viganò et al., 2013).

## Discussion

We have identified that OPCs acquire K_V_ and Na_V_ channels, AMPA/KARs, and NMDARs during development. The time of onset, peak expression, and decline of these ion channels seems to be determined by environmental factors rather than being solely regulated by intrinsic mechanisms or developmental site of origin because the most significant changes are detected when OPCs from all developmental sites of origin are present at similar proportions (Kessaris et al., 2006). These findings are consistent with recent studies of origin-mapped OPCs (Marques et al., 2018, Tripathi et al., 2011). The most prominent age-related changes we observed were those related to Na_V_ channels (which determines OPC excitability) and NMDAR densities, which both peaked at a time when OPCs start to differentiate into myelinating oligodendrocytes and begin to express myelin genes (Figures 1 and 2; Marques et al., 2018). At this time, OPCs are highly sensitive to changes in neuronal activity, and this sensitivity changes differently with age depending on the region. As the myelination potential of OPCs declines, so do their NMDAR and Na_V_ densities and, thus, their excitability; intriguingly, NMDARs disappear earliest in a non-myelinating brain region but remained present throughout life in OPCs in a neurogenic region.

We have shown previously that NMDARs are important for activity-dependent myelination (Gautier et al., 2015, Lundgaard et al., 2013), but only when NMDARs are pre-activated in the presence of the growth factors neuregulin or brain-derived neurotrophic factor (BDNF), to switch OPCs to an activity-dependent myelination mode. In the absence of pre-activation, myelination occurs independent of neuronal activity but at a slower rate (Lundgaard et al., 2013). Accordingly, developmental myelination is slowed down (Kougioumtzidou et al., 2017, Saab et al., 2016) but relatively unaffected by knockout of NMDARs or AMPARs in oligodendrocyte-lineage cells (De Biase et al., 2011, Kougioumtzidou et al., 2017, Saab et al., 2016). This might be due to the fact that when receptors are knocked out of OPCs before they are expressed and activated, myelination occurs by default, independent of neuronal activity and glutamate receptor activation. However, presumably, when AMPA/KAR and NMDAR expression has started, and OPCs have been switched to depend on NMDAR activation for myelination, a loss of NMDARs, such as with age, may impede OPCs from terminal differentiation into myelinating oligodendrocytes. When culturing OPCs from this time point in myelination-promoting medium, they did not myelinate, similar to when NMDARs are blocked after they are expressed and activated, myelination is reduced (Gautier et al., 2015, Lundgaard et al., 2013).

The supposedly defining feature of OPCs is the expression of Na_V_, and OPCs that lack these channels have been suggested to be at the starting point of differentiation. However, we show here that OPCs are born without Na_V_ and that the proportion of OPCs with these channels increases during the first postnatal weeks and then stays relatively constant throughout the ages tested. We identified that OPCs in an active cell cycle have a higher density of Na_V_. Concomitantly, Na_V_ density peaks when OPC proliferation is highest and decreases in line with the decrease in the proportion of OPCs in G2/M phase and expression of proliferation genes as well as NMDAR density and myelin capacity. In contrast, AMPA/KAR densities increase with age and are expressed in all OPCs after birth. The tight control of AMPA/KAR, NMDA, and Na_V_ densities we observed was unexpected, and so was the seemingly temporally opposing expression of AMPA/KARs and NMDARs in the parenchymal areas, similar to when NMDARs are knocked out of OPCs, AMPAR densities increase (De Biase et al., 2011). AMPARs have been reported to moderate OPC survival and promote more efficient myelination at a time when NMDARs are present (Kougioumtzidou et al., 2017). In line with this, we have previously found that AMPARs are important for the early stages of the remyelination process, whereas NMDAR activation is important for the later stages (Gautier et al., 2015, Lundgaard et al., 2013).

It is important to note that, at all time points studied and in all regions, OPCs with embryonic electrophysiological properties were identified, but their proportion differed, with the highest proportion through all ages being found in the SVZ. The proportion of OPCs with different displays of ion channels and the density of each channel differed with age and region. The heterogeneity identified here may therefore reflect different cellular states, where densities of ion channels define a particular functional state of OPCs.

For example, when OPCs first appear, at E13, they have no detectable voltage-gated ion channels or glutamate receptors and may therefore be considered as being in a naive state. The first ion channels to appear are K_V_ and AMPA/KARs, at E18. OPCs with these properties may indicate a migratory cell state because we identified a strong expression of migratory genes that then declined postnatally at this time point. The fraction of OPCs with detectable Na_V_ and the Na_V_ density increase sharply around birth. It is conceivable that OPCs with high Na_V_ and K_V_ and low AMPA/KAR densities reflect a high proliferation state of OPCs because (1) OPCs in S/G2/M phase have a higher density of Na_V_ than OPCs in G_0_/G1 phase (Figure 3H), (2) the proportion of this type of OPC is the most prominent (Figure 1) at the time when the highest proportion of OPCs are in G2/M phase (Figure 3E), (3) EdU-positive OPCs show this pattern of ion channel expression (Clarke et al., 2012), and (4) this state of OPCs is the most prominent during the OPC recruitment phase (the period of highest proliferation) during myelin regeneration (Gautier et al., 2015). NMDARs are the last to appear at P0 and the only ion channel that disappears within the age range tested. OPCs expressing Na_V_, K_V_, AMPA/KARs, and NMDARs are most prominent at the time of OPC differentiation during development, when myelin gene expression starts (Figure 2; Marques et al., 2018), and at the start of the differentiation phase of myelin regeneration (Gautier et al., 2015) and so may reflect a “primed” OPC state for differentiation. K_V_ and AMPA/KAR channels were expressed in nearly all recorded postnatal OPCs. Intriguingly, not all OPCs have Na_V_ or NMDARs, but their channel density reaches a maximum after the first postnatal week, at a time when myelination starts and myelin-related genes start to be detected in OPCs, and declines again when the rate of myelination declines and both cell cycle and differentiation gene expression decline. The last state of OPCs represents low Na_V_ density, lack of NMDARs, and high AMPA/KAR density and is observed at a time when OPC cell-cycle time lengthens, cell-cycle and differentiation genes are downregulated and senescent molecular signature genes appear, and OPCs differentiation potential declines. Thus, perhaps this state represents a “quiescent” state. Although we could not differentiate between G1 and G_0_, the fact that there was a significant difference in variance in AMPA/KAR density (and not in Na_V_ or NMDAR densities) in cells recorded in G_0_/G1 phase suggests that AMPA/KAR may differ between G1 and G_0_.

It is possible that these OPC states reflect prior identified subtypes of OPCs (Marques et al., 2016) or different epigenetic states (Liu et al., 2016). Currently, it is not clear whether the detected changes in Na_V_ channel, AMPA/KAR, and NMDAR densities drive the age-related changes in OPC behavior nor whether maintaining high densities of NMDARs and Na_V_, and low AMPA/KAR densities in OPCs would counteract the age-dependent decline in myelination. What is clear is that these changes in Na_V_ channel, AMPA/KAR, and NMDAR densities will have a profound effect on how OPCs sense neuronal activity and on the effect neuronal inputs will have on OPCs. With increasing understanding of the regulation of gene expression and generation of novel biological engineering technology, it may become possible to manipulate Na_V_ channel, AMPA/KAR, and NMDAR densities in OPCs postnatally. However, as postnatal Na_V_ channels, AMPA/KAR and NMDAR gene expression is relatively stable; this indicates more that the detected changes in the density of these channels are post-transcriptionally and/or post-translationally regulated. How these changes are regulated is currently unknown, but presumably they are environmentally regulated (Figure S3) although seemingly not easily reversible (Figure 5). How the environment regulates these changes is not known. G protein-coupled receptors such as metabotropic glutamate receptors (mGluRs) and growth factors have been shown to regulate glutamate receptor expression in OPCs (Gallo et al., 1994, Lundgaard et al., 2013, Zonouzi et al., 2011). Moreover, cytokines have been shown to alter AMPAR densities in hippocampal neurons (Stellwagen and Malenka, 2006), and here we identified that immune system genes, including cytokine receptors, were upregulated in old OPCs compared with young OPCs. Accordingly, a combination of G-protein-coupled receptors, growth factors, and cytokines may underlie these changes.

In summary, our data show that OPCs are functionally heterogeneous between regions and within regions at any given time point tested. These findings provide evidence of the functional relevance of heterogeneity identified at the transcriptional level. This heterogeneity in physiological properties may underlie the myelination potential of OPCs and implicate different functions or cell states.

## STAR★Methods

### Key Resources Table

**Table:** 

REAGENT or RESOURCE	SOURCE	IDENTIFIER
**Antibodies**		

Anti-NG2 Chondroitin Sulfate Proteoglycan Antibody	Millipore	Cat# MAB5384; RRID: AB_177646
Anti-GFP antibody	Abcam	Cat# ab13970; RRID: AB_300798
Anti-Ki67 antibody [SP6]	Abcam	Cat# ab16667; RRID: AB_302459
Anti-mCherry antibody	Abcam	Cat# ab167453; RRID: AB_2571870
CDP Antibody (M-222) (Cux1)	Santa Cruz Biotechnology	Cat# sc-13024; RRID: AB_2261231
Anti-Ctip2 antibody [25B6] - ChIP Grade	Abcam	Cat# ab18465; RRID: AB_2064130
Doublecortin Antibody (C-18)	Santa Cruz Biotechnology	Cat# sc-8066; RRID: AB_2088494
Rat anti MBP (aa82-87)	Bio-Rad	Cat# MCA409S; RRID: AB_325004
Monoclonal Anti-Neurofilament 160/200 antibody produced in mouse	Sigma-Aldrich	Cat# N2912; RRID: AB_477262
Alexa Fluor 647 Mouse anti-Ki-67 (Clone B56)	BD PharMingen	Cat# 558615; RRID: AB_647130
Goat Anti-Chicken IgY H&L (Alexa Fluor 488)	Abcam	Cat# ab150169; RRID: AB_2636803
Goat Anti-Chicken IgY H&L (Alexa Fluor 568)	Abcam	Cat# ab175477
Alexa Fluor 647 AffiniPure Donkey Anti-Chicken IgY (IgG) (H+L)	Jackson ImmunoResearch Labs	Cat# 703-605-155; RRID: AB_2340379
Invitrogen Goat anti-Rabbit IgG (H+L) Highly Cross-Adsorbed Secondary Antibody, Alexa Fluor 647	ThermoFisher	Cat# A-21245; RRID: AB_2535813
Goat anti-Rat IgG (H+L) Cross-Adsorbed Secondary Antibody, Alexa Fluor 647	ThermoFisher	Cat# A-21247; RRID: AB_141778
Goat anti-Rat IgG (H+L) Cross-Adsorbed Secondary Antibody, Alexa Fluor 488	ThermoFisher	Cat# A-11006; RRID: AB_2534074
Goat anti-Mouse IgG (H+L) Highly Cross-Adsorbed Secondary Antibody, Alexa Fluor 555	ThermoFisher	Cat# A-21424; RRID: AB_141780
Anti-Goat IgG (H+L), highly cross-adsorbed, CF 568 antibody produced in donkey	Sigma-Aldrich	Cat# SAB4600074

**Chemicals, Peptides, and Recombinant Proteins**		

DAPI	Sigma-Aldrich	Cat# D9542
Hoescht-33342	Thermo Fisher	Cat# H3570
Kainic Acid	Tocris	Cat# 0222
NMDA	Tocris	Cat# 0114
Strychnine hydrochloride	Sigma-Aldrich	Cat# S8753
Glycine	Sigma-Aldrich	Cat# G8898
Barium chloride dihydrate	Sigma-Aldrich	Cat# B0750
NaCl	Sigma-Aldrich	Cat# S7653
KCl	Sigma-Aldrich	Cat# P3911
NaHCO_3_	Sigma-Aldrich	Cat# S5761
NaH_2_PO_4_	Fisher Scientific	Cat# S/3760/53
CaCl_2_	VWR	Cat# 21114
MgCl_2_	Fisher Scientific	Cat# 15656060
D-glucose	Sigma-Aldrich	Cat# G7528
Kynurenic acid	Sigma-Aldrich	Cat# K3375
HEPES	Sigma-Aldrich	Cat# H3375
BAPTA	Sigma-Aldrich	Cat# A4926
D-gluconic acid	Sigma-Aldrich	Cat# G1951
CsOH	Sigma-Aldrich	Cat# 516988
KOH	Sigma-Aldrich	Cat# P5958
MgATP	Sigma-Aldrich	Cat# A9187
Na_2_GTP	Sigma-Aldrich	Cat# G8877
K-Lucifer Yellow	Sigma-Aldrich	Cat# L0144
rGDF11	PeproTech	Cat# 120-11
Fixation Buffer	BioLegend	Cat# 420801
Intracellular Staining Permeabilization Wash Buffer	BioLegend	Cat# 421002
Papain from papaya latex	Sigma-Aldrich	Cat# P3125
L-cysteine	Sigma-Aldrich	Cat# 30089
Deoxyribonuclease I from bovine pancreas, Type IV	Sigma-Aldrich	Cat# D5025
Albumine from bovine serum	Sigma-Aldrich	Cat# A4919
Trypsin inhibitor from Glycine max (soybean)	Sigma-Aldrich	Cat# T9003
Goat serum	Sigma-Aldrich	Cat# G9023
Donkey serum	Sigma-Aldrich	Cat# D9663
Triton X-100	Fisher BioReagents	Cat# BP151
Paraformaldehyde	Fisher Scientific	Cat# P/0840/53

**Critical Commercial Assays**		

Myelin Removal Beads II, human, mouse, rat	Miltenyi Biotec	Cat# 130-096-733
CD140a (PDGFRα) MicroBead Kit, mouse	Miltenyi Biotec	Cat# 130-101-502
RNAeasy Micro Kit	QIAGEN	Cat# 74004
SMARTer Stranded Total RNA-Seq Kit v2 – Pico Input Mammalian	Takara Clontech	Cat# 634411

**Deposited Data**		

Raw data files for RNA sequencing	This manuscript	GEO: GSE121083
Raw data files for Flow cytometry	This manuscript	https://flowrepository.org/id/RvFrXKqGrE6oSqbz3pUcrGHhBsNIcmlH5nBR0azMBaDS8vTjRXww0B6IbjNgEFkr

**Experimental Models: Organisms/Strains**		

Mouse: NG2-EYFP	Prof Jacqueline Trotter; Karram et al., 2008	N/A
Mouse: Fucci2a	Mort et al., 2014	IMSR Cat# RBRC06511, RRID: IMSR_RBRC06511
Mouse: STOCK Mki67tm1.1Cle/J	Basak et al., 2014	IMSR Cat# JAX 029802, RRID: IMSR_JAX:029802
Mouse: C56BL/6 wild-type	Charles River Laboratories	C57BL/6NCrl, RRID: IMSR_CRL:27

**Software and Algorithms**		

ImageJ	NIH	https://fiji.sc/ or https://imagej.nih.gov/ij/
LAS AF/LAS X	Leica	https://www.leica-microsystems.com/products/microscope-software/details/product/leica-las-x-ls/
MATLAB	MathWorks	https://uk.mathworks.com/
Cell capacitance analysis	Written in house	N/A
Na_V_ current analysis	Written in house	N/A
K+ conductance analysis	Written in house	N/A
SPSS	IBM	https://www.ibm.com/analytics/spss-statistics-software
GraphPad Prism	GraphPad Software	https://www.graphpad.com/scientific-software/prism/
TrimGalore!	Babraham Bioinformatics	https://www.bioinformatics.babraham.ac.uk/projects/trim_galore/
TopHat 2.1.1	Center for Computational Biology at Johns Hopkins University	https://ccb.jhu.edu/software/tophat/index.shtml
R Bioconductor DESeq2	Love et al., 2014	https://bioconductor.org/packages/release/bioc/html/DESeq2.html
DAVID	Laboratory of Human Retrovirology and Immunoinformatics	https://david.ncifcrf.gov/
REVIGO	Supek et al., 2011	http://revigo.irb.hr
FCS Express 6 Flow	De Novo Software	https://www.denovosoftware.com/site/Flow-RUO-Overview.shtml

### Contact for Reagent and Resource Sharing

Further information and requests for resources and reagents should be directed to, and will be fulfilled by, the Lead Contact, Ragnhildur T. Káradóttir (rk385@cam.ac.uk).

### Experimental Model and Subject Details

Experiments were performed in accordance with EU guidelines for the care and use of laboratory animals, and with the guidelines of the UK Animals (Scientific Procedures) Act 1986 and subsequent amendments. Use of animals in this project was approved by the Animal Welfare and Ethical Review Body for the University of Cambridge and carried out under the terms of UK Home Office Licenses P9B1FBC4B and 70/7715. All mice were maintained under a 14 h light:10 h dark cycle with food and water supplied *ad libitum*. For electrophysiological studies and cell cycle studies, mice were heterozygous knock-in mice (Karram et al., 2008) expressing EYFP under the endogenous NG2 promoter, kindly provided by Jacqueline Trotter, allowing for identification of OPCs by EYFP expression. The ages of mice used were from E13 to P503 as stated in text and figures. These mice were bred on a C57BL/6 background. To assess electrophysiological properties of different cell cycle states in OPCs we crossed Fucci2a mice, which labels different cell cycle phases (Mort et al., 2014), or Ki67-RFP mice (Basak et al., 2014) to the NG2-EYFP mice to generate NG2-EYFP:FUCCI and NG2-EYFP:Ki67-RFP mouse lines. Fucci2a mice were bred on a mixed CBA/Ca and C57BL/6 background, while Ki67-RFP mice were bred on a C57BL/6 background. For all three lines, both male and female mice were used. For flow cytometry experiments, male and female C57BL/6 NG2-EYFP mice were used. For OPC transcriptome analysis C57BL/6 wild-type mice (Charles River Laboratories) were used; all mice were female, except for the embryonic and P12 time points, where full litters were used and equal number of animals per sex was assumed. The mice for the GDF11 infusion experiment were all male C57BL/6 NG2-EYFP mice.

### Method Details

#### Brain slices

Acute brain slices (225 μm thick) were prepared either from the cerebellum (parasagittally cut) or the forebrain (coronally cut) from transgenic mice in ice-cold (∼1°C) oxygenated (95% O_2_–5% CO_2_) aCSF solution and kept at RT after a 1h recovery period. aCSF contained (in mM): 126 NaCl, 24 NaHCO_3_, 1 NaH_2_PO_4_, 2.5 KCl, 2.5 CaCl_2_, 2 MgCl_2_, 10 D-glucose, pH 7.4. 1mM Kynurenic acid was added to block glutamate receptors, which might be activated during the dissection procedure. For embryonic acute brain slices, the brains were embedded in agarose and sliced coronally in 225 μm thick sections as above.

#### Solutions

Slices were superfused at 22 ± 1°C with HEPES-buffered external solution containing (in mM): 144 NaCl, 2.5 KCl, 10 HEPES, 1 NaH_2_PO_4_, 2.5 CaCl_2_, 10 glucose, 0.1 glycine (to coactivate NMDARs), 0.005 strychnine (to block glycine receptors), 200μM BaCl_2_ (only in some instances to block potassium conductance), pH set to 7.4 with NaOH, continuously bubbled with 100% O_2_. OPCs were whole-cell clamped with electrodes containing a recording solution that comprised (in mM) of either 130 K-gluconate or 130 Cs-gluconate, 4 NaCl, 0.5 CaCl_2_, 10 HEPES, 10 BAPTA, 4 MgATP, 0.5 Na_2_GTP, 2 K-Lucifer yellow, pH set to 7.3 with KOH (or with CsOH). Final osmolarity was ∼290mOsm. For activation of glutamate receptors, KA 30 μM (activates both AMPA and KA receptor; Tocris) or NMDA 60 μM (Tocris) was bath applied.

#### Electrophysiology

For whole-cell patch-clamp experiments parenchymal EYFP positive OPCs were selected in the brain area of interest (EYFP positive cells located close to or on blood vessels were excluded). In embryonic slices, OPCs were selected in the developing CTX at a sufficient distance from nearby blood vessels. When unspecified CTX OPCs were mainly recorded in cortical layers 5-2. When unspecified, OPCs in the CC were in and around the anterior CC. Patched cells were confirmed as OPCs by their post-recording dye-fill morphology, which overlaid EYFP, and by post hoc antibody labeling against the proteoglycan NG2 to identify oligodendrocyte progenitors (106/107 tested labeled for NG2 (Millipore) or GFP/EYFP (Abcam)). For identification of OPCs location: CTX Layer 1 was identified as the area between the edge of the slice and layer 2 marked with Cux1 antibody labeling; CTX layer 5/6 was marked with CTIP2 immunoreactivity; dorsal SVZ was identified with doublecortin labeling and the adjacent ventricle; anterior CC was identified at bregma 0 and posterior CC was identified overlaying the hippocampus (Figures S4A–S4D). Recording electrodes had a resistance of 5–9 MΩ and the uncompensated series resistance was 40 ± 1 MΩ. Inclusion criteria was based on series resistance, leak current being lower than 500pA and a stable baseline. Electrode junction potential (−14 mV) was compensated. A Multiclamp 700B (Molecular Devices) or Axopatch 200 (Molecular Devices) was used for voltage clamp data acquisition. Data were sampled at 50 kHz and filtered at 10 kHz using pClamp10.3 (Molecular Devices).

#### Electrophysiological analysis

Synaptic events were detected based on threshold (amplitude > 2xSD) and included when decay time τ (10%–90%) was longer than its rise time. For event detection and analysis of frequency, amplitude and decay time τ pClamp 10.3 (Molecular Devices) and the Strathclyde Electrophysiology Software package WinEDR V3.3.7 WinWCP V4.6.2 were used. Voltage-gated ion channels, series resistance, and membrane capacitance was analyzed using custom-written MATLAB scripts (MathWorks).

#### GDF11 administration

EYFP-NG2 knock-in mice were implanted intraperitoneally with osmotic minipumps (Alzet Micro-Osmotic Pumps, model 1004, DURECT Corporation) at P150 for continuous i.p. delivery at a flow rate of 0.11 μl/h containing GDF11 (0.1mg/kg/d; rGDF11, catalog # 120-11, PeproTech) or saline. After 4-weeks delivery mice were used for electrophysiological recordings, only cells responding to an agonist were used for comparisons, the proportion of cells responding did not differ between conditions. The use of minipumps ensured reduced stress responses of the animals and consistent dosing compared to i.p. injections which require daily handling.

#### Cocultures

Mouse OPCs were isolated from EYFP-NG2 knock-in mice at different ages, as stated in manuscript, using magnetic cell sorting (MACS, Miltenyi Biotec). First myelin debris were removed from single-cell suspension by myelin removal beads (#130-096-733, Miltenyi), and subsequently, OPCs were isolated using microbeads conjugated to monoclonal PDGFRα (#130-101-502, Miltenyi). Myelinating co-cultures were made as previously described (Lundgaard et al., 2013). Briefly, dorsal root ganglion neurons were derived from E14–E16 rats and allowed to grow for 2 weeks before OPCs were plated on top. Myelination was analyzed after 3 weeks of co-culture in a non-blinded manner. Coverslips were imaged with an epifluorescence microscope and 3-4 images (depending on cell density) were randomly taken in each quadrant of the coverslip, with replicates of at least 4 coverslips. On coverslips where less than 5 MBP+ cells were detected (as in some coverslips carrying co-cultures with ‘old’ OPCs), each MBP+ cell was imaged individually. All MBP+ cells on the coverslip were counted and compared between ages (young: < P7; old: 8-24months). Cells were considered myelinating when there was a clear ensheathment of axons in an area around the cell soma. Cells merely attached to axons without elongated myelin sheaths in nearby axons or cells which produced sheaths without wrapping them around axons were considered as non-myelinating.

#### mRNA-seq libraries

All the mice used were wild-type C57BL/6 mice (Charles River Laboratories) at the ages detailed in the manuscript. OPCs were isolated as described above for co-cultures, except from the E15.5 and P12 time points, where the myelin removal step was omitted. For the E15.5 and P12 time-points, each sample (n = 3) was a pool of 2-3 brains, all other time-points were isolated from one brain per sample (n = 3, n = 4 for P306-308). After OPC enrichment RNA was extracted using RNeasy Micro Kit (QIAGEN) according to manufacturer’s instructions. The library was prepared with SMARTer Stranded Total RNA-Seq Kit v2 - Pico Input Mammalian (Takara Clontech) according to manufacturer’s instructions. Data was read on a HiSeq4000 a total of > 350M 150 bp strand-specific paired-end reads were generated.

#### Next-generation sequence data analysis

RNA-seq reads were pre-processed and quality-trimmed using *Trim Galore!* https://www.bioinformatics.babraham.ac.uk/projects/trim_galore). Reads were aligned to the mouse reference genome GRCm38/mm10 using *Tophat* (version 2.1.1, https://ccb.jhu.edu/software/tophat/index.shtml) allowing for one mismatch and selecting uniquely mapping reads only. Normalization was performed and differential expression of genes was statistically evaluated by using the R Bioconductor DESeq2 package (https://bioconductor.org/packages/release/bioc/html/DESeq2.html).

Gene ontology (GO) term enrichment analysis for differentially expressed genes was performed using DAVID (https://david.ncifcrf.gov). GO terms were represented according to their semantic similarity using REVIGO (http://revigo.irb.hr).

#### Flow cytometry

NG2-EYFP mice were used for cell cycle analysis in the NG2 population, at the ages detailed in the manuscript. For animals older than three weeks, myelin debris were removed from single-cell suspensions with myelin removal beads (#130-096-733, Miltenyi Biotec) according to manufacturer’s instructions. Cells were fixed in Fixation Buffer (BioLegend) for 15 min, permeabilized with Intracellular Staining Permeabilization Wash Buffer (BioLegend) for 10 min. After washing, cells were stained with DAPI (1μg/ml) for 10 min. Cells were washed again and acquired on a BD LSRFortessa flow cytometer (BD Bioscience) equipped with four lasers, a blue (488-nm), a red (640-nm), a violet (405-nm) and a yellow/green (561-nm) laser. The EYFP positive cells were detected by filtration through a 530/30 nm band pass (BP) filter and DAPI through a 450/50 nm BP filter. Analysis was performed with FCS Express 6 Flow (*De Novo* Software) (Figure S2).

#### Immunohistochemistry

225μm thick slices were cut as described above, and fixed in 4% paraformaldehyde. Antibody labeling was performed as described previously (Káradóttir and Attwell, 2006). Briefly, slices were incubated in permeabilization and blocking solution (10% goat or donkey serum, 0.5% Triton X-100, in PBS) for 4-6 hours. Slices were incubated with primary antibodies, in PBS, overnight. After washing, slices were incubated with secondary antibodies, in PBS, overnight at 4°C. Following washing and DAPI staining, slices were mounted and imaged on a Leica SP5 confocal microscope.

To measure proliferation with immunohistochemistry, 3-6 slices from an average of 2 animals were quantified for each time point. Images were taken on a confocal microscope. 6 z stacks (3 in the cortex and 3 in the CC) were taken randomly in each slice. Z stacks were taken in order to include all EYFP+ cells in a field of view. Analysis was performed manually on the maximum projection of each stack, with the number of EYFP+ Ki67+ cells divided by the number of EYFP+ cells. Experimenters were not blind to age during quantification. All data collected was included in the statistical analysis, which was performed with a one-way ANOVA for each brain region.

### Quantification and Statistical Analysis

All statistical analysis was performed in GraphPad Prism or SPSS software or manually calculated. Data are shown as mean ± s.e.m. For electrophysiology results, numbers of cells are indicated on bars. For flow cytometry analysis (Figure 3), number of animals are indicated on bars. Individual coculture numbers are indicated on the bar graph for coculture experiments (Figure 5). When relevant, normality of data was assessed using Shapiro–Wilk tests. Non-parametric tests that do not assume data follow a normal distribution gave the same conclusions for significant and non-significant differences in all cases. Significance in the variance between dataset was assessed using F-test or Brown–Forsythe test. One-way ANOVA, or Welch’s ANOVA when variances were unequal, followed by Holm–Bonferroni-corrected post hoc t tests were used to compare multiple samples. Other P values for comparison of means are from Student’s two-tailed t tests, with Welch’s correction when variances were unequal. Differences in percentages between age groups were analyzed by Chi-square test with Yates’s correction for small n-numbers.

### Data and Software Availability

#### Next-generation sequence data

All data have been deposited in NCBI’s Gene Expression Omnibus (Edgar et al., 2002) and are accessible through GEO Series accession number GSE121083 (https://www.ncbi.nlm.nih.gov/geo/query/acc.cgi?acc=GSE121083).

#### Flow cytometry data

All flow cytometry data has been deposited in Flowrepository.org (https://flowrepository.org/id/RvFrXKqGrE6oSqbz3pUcrGHhBsNIcmlH5nBR0azMBaDS8vTjRXww0B6IbjNgEFkr)

#### Electrophysiology analysis scripts

Cell capacitance, Na_V_ current_,_ K_V_ current and K^+^ conductance analysis scripts are available upon request.

## Supplemental Information

Document S1. Figures S1–S4

Table S1. OPC mRNA Expression across Age and GO Term Analysis, Related to Figure 2

Document S2. Article plus Supplemental Information
