# Supplementary material for: Oligodendrocyte Progenitor Cells Become Regionally Diverse and Heterogeneous with Age
Source: Neuron. 2019 Feb 6;101(3):459–471.e5. doi: 10.1016/j.neuron.2018.12.020 (PMC6372724; doi:10.1016/j.neuron.2018.12.020)
Supplement: Document S1. Figures S1–S4 [file mmc1.pdf]

Neuron, Volume *101*

Supplemental Information

Oligodendrocyte Progenitor Cells Become Regionally Diverse and Heterogeneous with Age

Sonia Olivia Spitzer, Sergey Sitnikov, Yasmine Kamen, Kimberley Anne Evans, Deborah Kronenberg-Versteeg, Sabine Dietmann, Omar de Faria Jr., Sylvia Agathou, and Ragnhildur Thóra Káradóttir

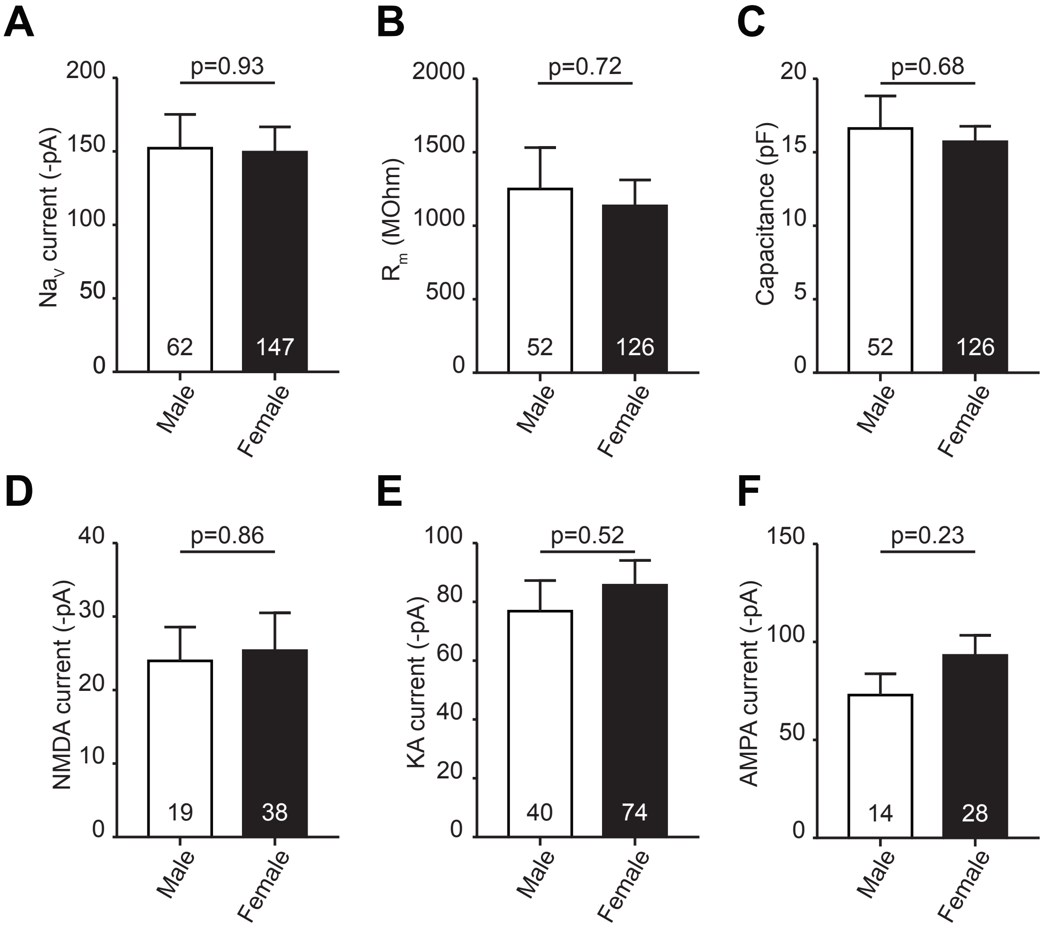

Figure S1. OPC membrane properties do not differ between sexes, related to Figure 1.

Average evoked currents in OPCs and intrinsic membrane properties such as membrane resistance (Rm) and capacitance (Cm; cell surface) do not differ between males and females. Cell numbers are shown on bar graphs from 41 female and 22 male mice. P values shown are from student t-test. Data is shown ±SEM.

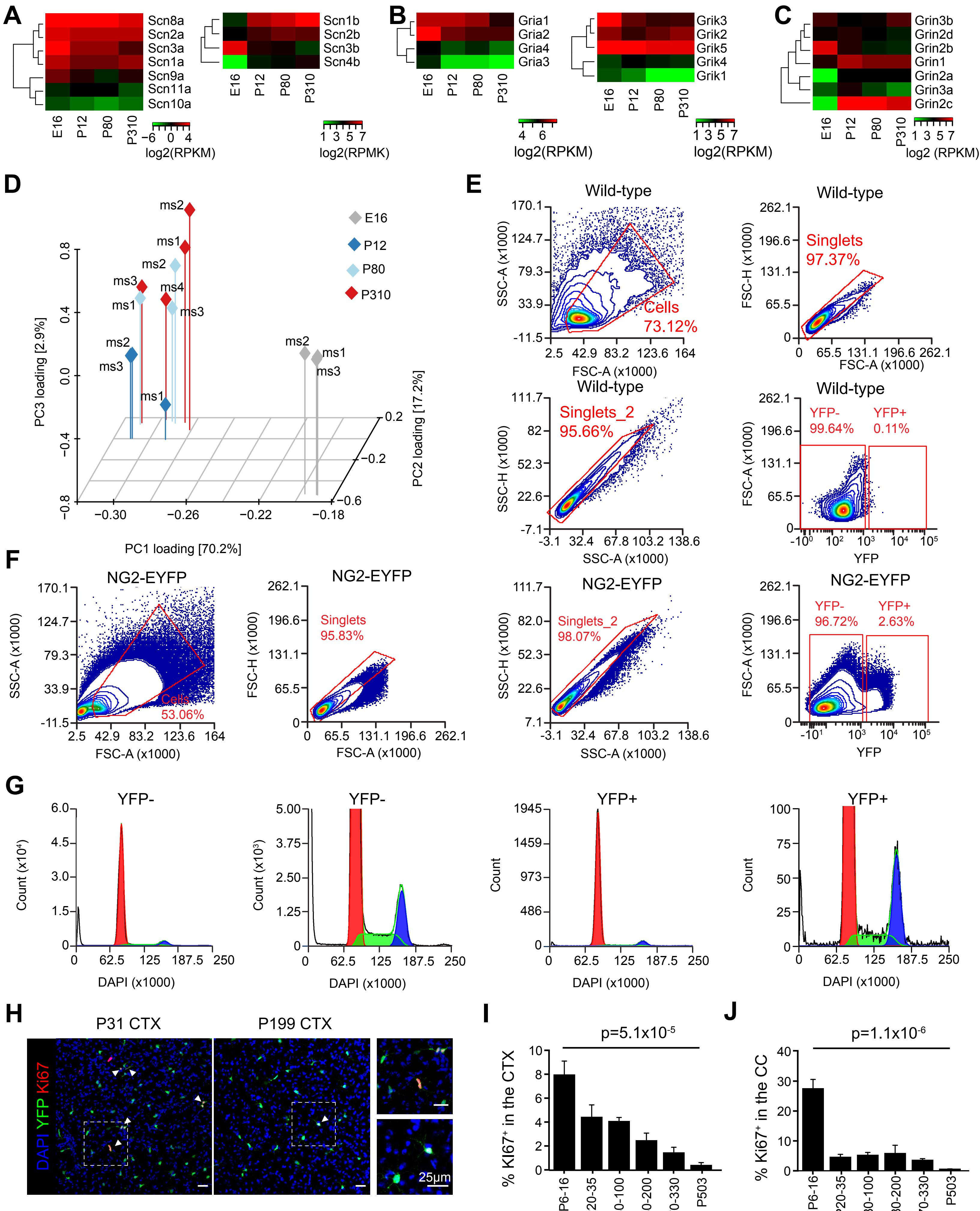

Supplementary Figure 2

Figure S2. Age related changes in OPCs ion channel expression, and cell cycle, related to Figure 2 and Figure 3.

(A-C) Expression pattern heat map of OPC Na_V α subunits and β auxiliary subunits (**A**), AMPA/KAR subunits (**B**), and NMDAR subunits (**C**) gene expression from embryonic (E16) to postnatal timepoints (P12, P80, P310). Reads per kilobase million (log2(RPKM)) are plotted for each gene.
(D) Principal component analysis (PCA) showing clustering of RNA-seq samples by age, per sample (some samples are pooled sorted OPCs from up to 3 animals).

(E-F) FACS plots showing the gating strategy used for cell cycle analysis of EYFP negative and EYFP positive cell populations by flow cytometry. Shown here are representative plots of a wild type mouse (**E**) and a NG2-EYFP mouse (**F**). The initial cell population was identified by gating based on forward versus side scatter (FSC vs SSC). Singlets were selected on the basis of FSC-A (area) and FSC-H (height), as well as SSC-A and SSC-H. Singlets present a relative distribution of size height versus area, whereas doublet cells present a higher area than height. Within the initial cell gate OPCs were defined as those positive for EYFP. Numbers indicate percentages in the gates.

(G) Cell cycle analysis by flow cytometry of EYFP negative and EYFP positive cell populations of a NG2-EYFP (P11) mouse. Two plots for each EYFP negative and EYFP positive populations are shown at two different magnifications to allow for better visualisation of the different cell cycle stages, identified by intensity of DAPI fluorescence. Colour coding of peaks representing different cell cycle stages.

(H) Immunohistochemistry for DAPI (blue), YFP (green) and Ki67 (red) to quantify the proportion of actively cycling OPCs across age. Ki67 positive OPCs (arrow heads) could be detected at all time points. Scale bar 25µm.

(I-J) The proportion of Ki67 positive OPCs declined both in the cortex (CTX) **(I)** and the corpus callosum (CC) **(J)** with age (CTX, $p=5.1x10^{-5}$, CC, $p=1.1x10^{-6}$, ANOVA). Analysis was performed on 3-6 cortical sections from an average of two animals.

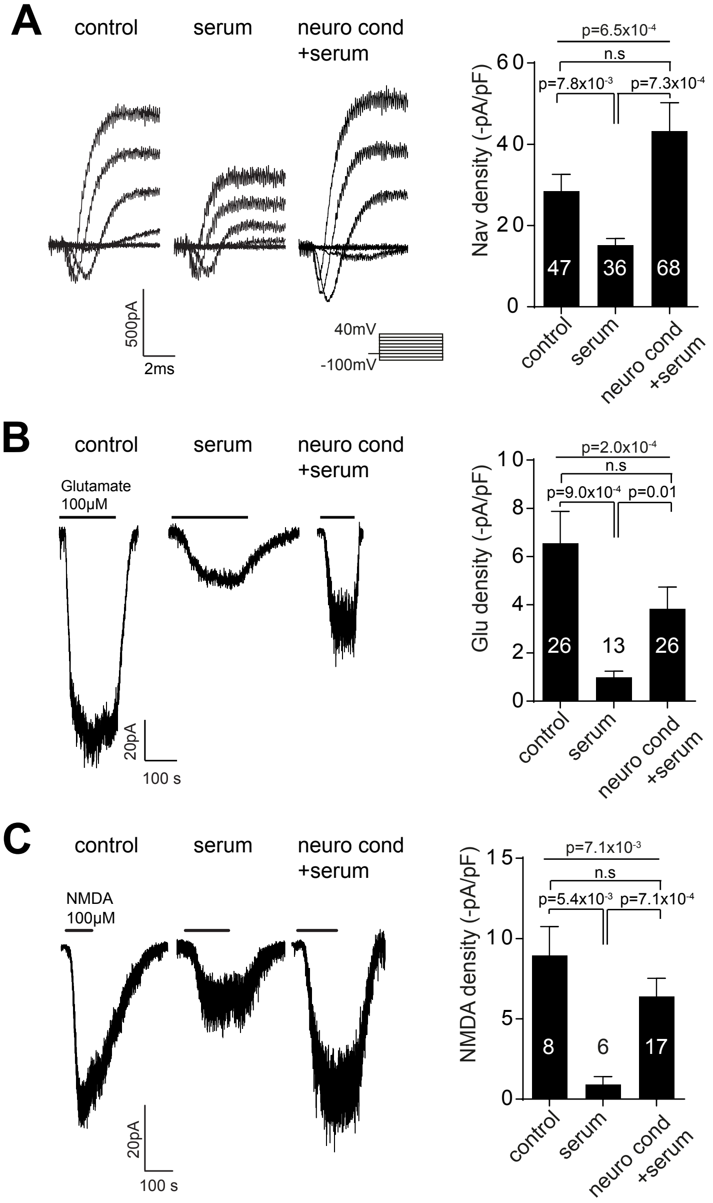

Figure S3.
The environment alters the functional ion channel density in neonatal OPCs, related to Figure 4 and 5.

(**A-C**) Whole-cell voltage clamp (V_h = -74mV) recorded currents in neonatal OPCs (P2) in vitro, cultured in different media (**A**) Na_V densities were significantly different between culture condition groups ($p=6.5x10^{-4}$, one-way ANOVA), serum decreased Na_V channel density in OPCs ($p=7.8x10^{-3}$), whereas neuronal conditioned media prevented changes mediated by foetal calf serum ($p=7.3x10^{-4}$), returning to similar densities as control (p=0.076, Holm-Bonferroni post-hoc test).

(**B**) The glutamate (100µM) evoked current density was significantly different between culture condition groups ($p=2x10^{-4}$, ANOVA). Foetal calf serum reduces glutamate evoked current density ($p=9.0x10^{-4}$) whereas neuronal conditioned media reverts changes mediated by foetal calf serum (p=0.011) to control densities (p=0.1, Holm-Bonferroni post-hoc test).

(**C**) The NMDA (60µM)-evoked current density was significantly different between culture condition groups ($p=7.1x10^{-3}$, one-way ANOVA). Foetal calf serum reduces NMDA evoked current density ($p=7.1x10^{-4}$) whereas neuronal conditioned media reverts changes mediated by foetal calf serum ($p=5.4x10^{-3}$) to control densities (p=0.26, Holm-Bonferroni post-hoc test).

N numbers on bar graphs represent cell numbers. Data is shown ±SEM.

Supplementary Figure 3

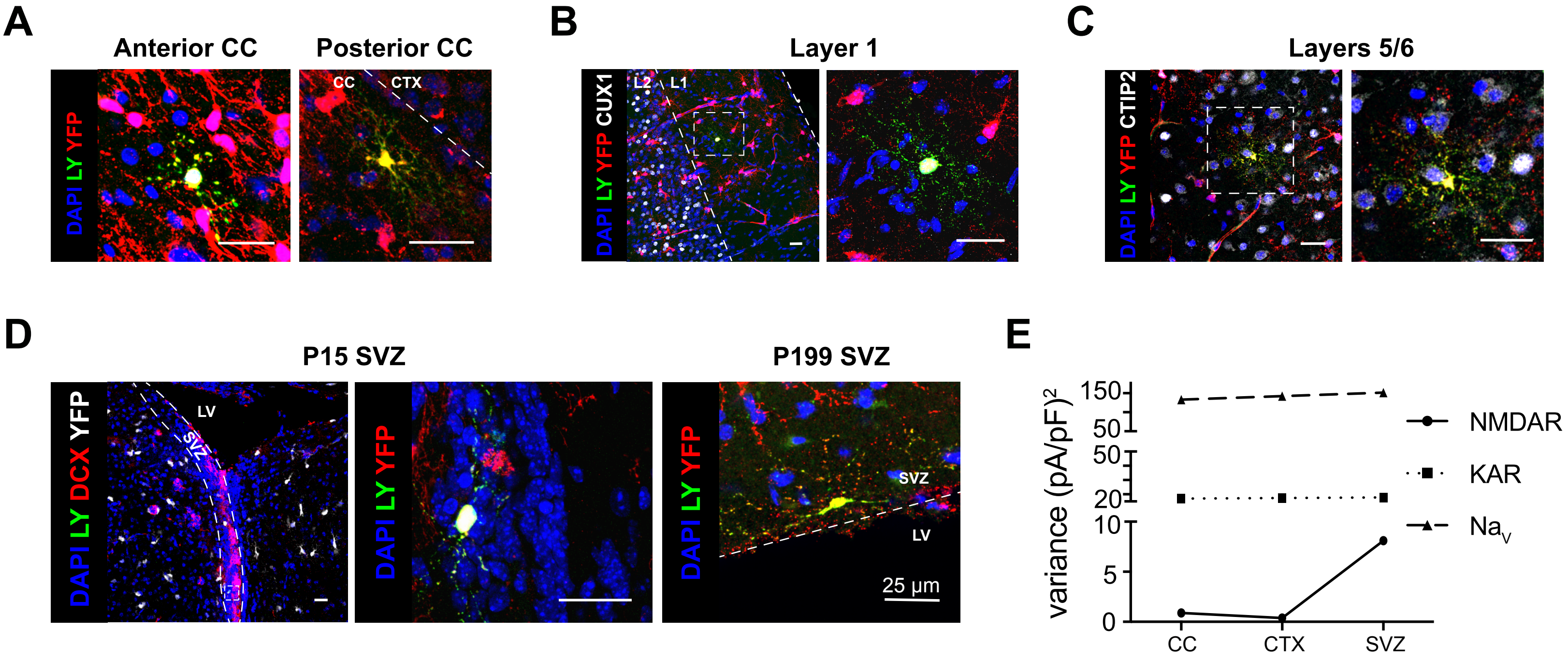

Figure S4. Identification of whole-cell patched OPCs in corpus callosum, cortex and subventricular zone, related to STAR Methods and Figure 6.

(**A**) EYFP positive cells were selected for whole-cell patch-clamp recording in the anterior (left) and posterior (right) corpus callosum. During recordings, cells were filled with Lucifer Yellow (LY; green), and EYFP expression (red) was confirmed with post-hoc immunohistochemistry. Scale bar 25µm.

(**B**) Cells were selected by their EYFP expression in layer 1 of the cortex, between the edge of the slice and layer 2. OPCs were dye-filled with Lucifer Yellow (LY; green) during whole-cell patch-clamp recording, and post hoc-labelled for YFP (red). Location verified by being above CUX1 immunoreactivity detecting layer 2 (white). Scale bar 25µm.

(**C**) Cell were selected by their EYFP expression in cortical layers 5 and 6 of the cortex. LY filled (green) cells were identified by proximity to the corpus callosum, as well as post-hoc immunohistochemistry against CTIP2 (white) and YFP (red). Scale bar 25µm.

(**D**) Cell were selected by their EYFP expression in and around the subventricular zone, in a densely populated region along the lateral ventricle. Post-hoc immunohistochemistry against DCX (red) was used to locate OPCs (LY filled and YFP positive) in the SVZ after recordings.

(**E**) The variance in receptor density (pA/pF)^2 in the corpus callosum, the cortex, and the subventricular zone. No difference was observed in NaV or KAR densities, but the variance in NMDAR density was significantly higher ($p<1 \times 10^{-15}$; Brown–Forsythe) in the SVZ compared to the corpus callosum or the cortex.
